# Home enteral nutrition for patients with esophageal cancer undergoing esophagectomy: A systematic review and meta-analysis

**DOI:** 10.3389/fnut.2022.895422

**Published:** 2022-07-28

**Authors:** Chi Zhang, Li-Wen Hu, Yong Qiang, Zhuang-Zhuang Cong, Chao Zheng, Wen-Feng Gu, Chao Luo, Kai Xie, Yi Shen

**Affiliations:** ^1^Department of Cardiothoracic Surgery, Medical School of Nanjing University, Jinling Hospital, Nanjing, China; ^2^Department of Cardiothoracic Surgery, School of Medicine, Jinling Hospital, Southeast University, Nanjing, China; ^3^Department of Cardiothoracic Surgery, School of Clinical Medicine, Jinling Hospital, Southern Medical University, Guangzhou, China; ^4^Department of Cardiothoracic Surgery, School of Clinical Medicine, Jinling Hospital, Nanjing Medical University, Nanjing, China

**Keywords:** HEN, home enteral nutrition, esophageal cancer, esophagectomy, nutritional status

## Abstract

**Introduction:**

Home enteral nutrition (HEN) is a relatively new nutritional intervention that provides patients with EN support at home through jejunostomy or nasogastric feeding tubes. We conducted this systematic review and meta-analysis to explore the safety and effect of HEN compared with normal oral diet (NOD) in postoperative patients with esophageal cancer (EC).

**Methods:**

EMBASE, Medline, Web of Science, and the Cochrane Library were used to search articles in English-language journals. The intervention effect was expressed using risk ratios (RRs) for dichotomous outcomes and mean differences (MDs) for continuous outcome measures, with 95% confidence intervals (95% CIs). The chi-square test and I-square test were used to test heterogeneity among studies.

**Results:**

Four studies were eventually included in this meta-analysis. Compared with NOD, HEN has a favorable impact on postoperative body mass index (BMI) (weighted mean difference [WMD] = 0.70, 95% CI: 0.09–1.30, *P* = 0.02), lean body mass (LBM) (WMD = 0.76, 95% CI: 0.04–1.48, *P* = 0.04), and appendicular skeletal muscle mass index (ASMI) (WMD = 0.30, 95% CI: 0.02–0.58, *P* = 0.03). Physical function (WMD = 9.26, 95% CI: 8.00–10.53, *P* < 0.001), role function (WMD = 9.96, 95% CI: 8.11–11.82, *P* < 0.001), and social function (WMD = 8.51, 95% CI: 3.48–13.54, *P* = 0.001) of the HEN group were better than those of the NOD group at 3 months, and HEN could reduce the fatigue of patients (WMD = −12.73, 95% CI: −14.8 to −10.66, *P* < 0.001) and the incidence of postoperative pneumonia (RR = 0.53, 95% CI: 0.34–0.81, *P* = 0.004). There was no significant difference in albumin between HEN and NOD groups (WMD = 0.05, 95% CI: −0.03 to 0.13, *P* = 0.20).

**Conclusion:**

HEN improved nutritional status and quality of life (QOL) in postoperative patients with EC and reduced fatigue and the incidence of postoperative pneumonia. All in all, the results of our meta-analysis support the use of HEN after esophagectomy.

## Introduction

Esophageal cancer (EC) is the sixth leading cause of cancer-related death worldwide, and its mortality is second only to gastric cancer in gastrointestinal tumors (1). Despite the advancement in therapies, including surgery, radiotherapy, and chemotherapy, the prognosis of patients with EC is still very poor (2).

More than half of these patients do not have enough oral intake when they are discharged from the hospital (3). Normal intake patterns may be changed after esophagectomy due to complications such as nausea and vomiting, pain, and dyspepsia. It takes 3–9 months for patients to regain a defined intake pattern after esophagectomy (4). Owing to insufficient oral intake coupled with high catabolic metabolism, patients are at a risk of severe malnutrition, which prolongs patients' postoperative recovery time and reduces their quality of life (QOL). Multiple studies have confirmed that the degree of malnutrition is positively correlated with the incidence of postoperative complications (5, 6). Therefore, providing adequate postoperative nutrition as early as possible is very important to reduce the severity of complications and improve the QOL of patients.

Compared with parenteral nutrition (PN), enteral nutrition (EN) support is more economical and more effective, with shorter hospital stays and fewer complications such as pneumonia (7, 8). Early EN has also been demonstrated to maintain the integrity of intestinal mucosa and immune function (9). Home enteral nutrition (HEN) has been proved to be an effective nutritional intervention since it was proposed in the 1970s. HEN refers to routine nutritional treatment after the operation, followed by EN through a jejunostomy tube or through a nasogastric feeding tube for more than 1 month after discharge on the basis of a normal oral diet (NOD). In 2019, ESPEN developed HEN guidelines advising patients at a risk of malnutrition, such as those with gastrointestinal or other malignant tumors, to consider oral nutrition supplements or HEN (10). However, this guideline does not elaborate on the effect of HEN in postoperative patients with EC. In addition, several randomized controlled trials have explored the relationship between HEN and the QOL or complications of patients after esophagectomy, but the results have not reached a consensus. Therefore, we conducted this systematic review and meta-analysis to explore the safety and effect of HEN compared with NOD in postoperative patients with EC.

## Methods

### Search strategy

Systematic literature retrieval of the EMBASE, Medline, Web of Science, and the Cochrane Library was performed up to date 1 February 2022, using the following search strategies and terms: (((((((esophagus [Title/Abstract]) OR esophageal [Title/Abstract]) OR esophagus [Title/Abstract]) OR esophageal [Title/Abstract])) AND (((tumor [Title/Abstract]) OR cancer [Title/Abstract]) OR carcinoma [Title/ Abstract])) AND (((home enteral nutrition [Title/Abstract]) OR home enteral feeding [Title/ Abstract]) OR HEN [Title/Abstract]))). Only articles published in English language were included.

### Selection criteria

The eligibility of studies was assessed by two independent reviewers by reviewing titles, abstracts, or full text identified by the search. Studies meeting the following criteria were included: (1) the patients in the study were diagnosed as EC histologically and underwent surgery; (2) studies assessed the effects of HEN and NOD on any of the following clinical endpoints: nutrition-related indicators, QOL, or complications; and (3) only the newest, largest, or most informative article was included if there were multiple articles based on similar populations. The exclusion criteria were listed as follows: (1) in animal experiments or *in vitro* studies; (2) case report, meta-analysis, review, editorial, and expert opinion; (3) without sufficient data for meta-analysis; and (4) duplicated studies.

### Data extraction and quality assessment

Two reviewers (Chi Zhang and Zhuangzhuang Cong) were involved to identify the eligible studies, and disagreements were resolved by a third reviewer (Chao Zheng). Characteristics (first author, publication year, country, number of patients, pathological and clinical data of patients, intervention, and duration) were extracted. Outcome measures, including nutrition-related indicators, QOL, and complications, were extracted from the text or tables of the included articles. Cochrane risk of bias tool including randomization sequence generation, allocation concealment, blinding, incomplete outcome data, selective reporting, and other biases, was used to assess the internal authenticity. The quality of evidence was evaluated using the Grading of Recommendations Assessment, Development and Evaluation (GRADE) system, which takes into account statistical heterogeneity, publication bias, risk of bias, indirectness, and statistical imprecision (11). The following system was used to interpretive scoring of the heterogeneity and downgrading of the evidence: *P* > 0.05, low heterogeneity; 0.01 < *P* ≤ 0.05, moderate heterogeneity, downgrade evidence by one level; P ≤ 0.01, high heterogeneity, downgrade evidence by two levels. An exception was that if visual inspection of forest plots showed the consistent direction of study-level effect estimates, the quality of evidence will only be downgraded by one level, even if the statistical heterogeneity was high. Our overall confidence in the reliability of the pooled data was rated from “very low,” “low,” “moderate,” to “high”.

### Statistical analysis

The intervention effect was expressed using risk ratios (RRs) for dichotomous outcomes and mean differences (MDs) for continuous outcome measures, with 95% confidence intervals (95% CIs). *P* < 0.05 were considered statistically significant. The chi-square test and I-square test were used to test heterogeneity among studies. An I^2^ > 50% or *P* < 0.1 was considered to indicate significant heterogeneity among studies. A random-effects model was selected if heterogeneity was identified among studies; otherwise, a fixed-effects model was used. In addition, if limited studies were included, a random-effects model of the DerSimonia and Laird method was used. Publication bias was evaluated by the funnel plot with Egger's weighted regression method and Begg's rank correlation method, and a *P* > 0.05 indicated no publication bias (12, 13). Analyses were performed using Stata version 15.0 (Stata Corporation, College Station, TX).

## Results

**Figure 1:**
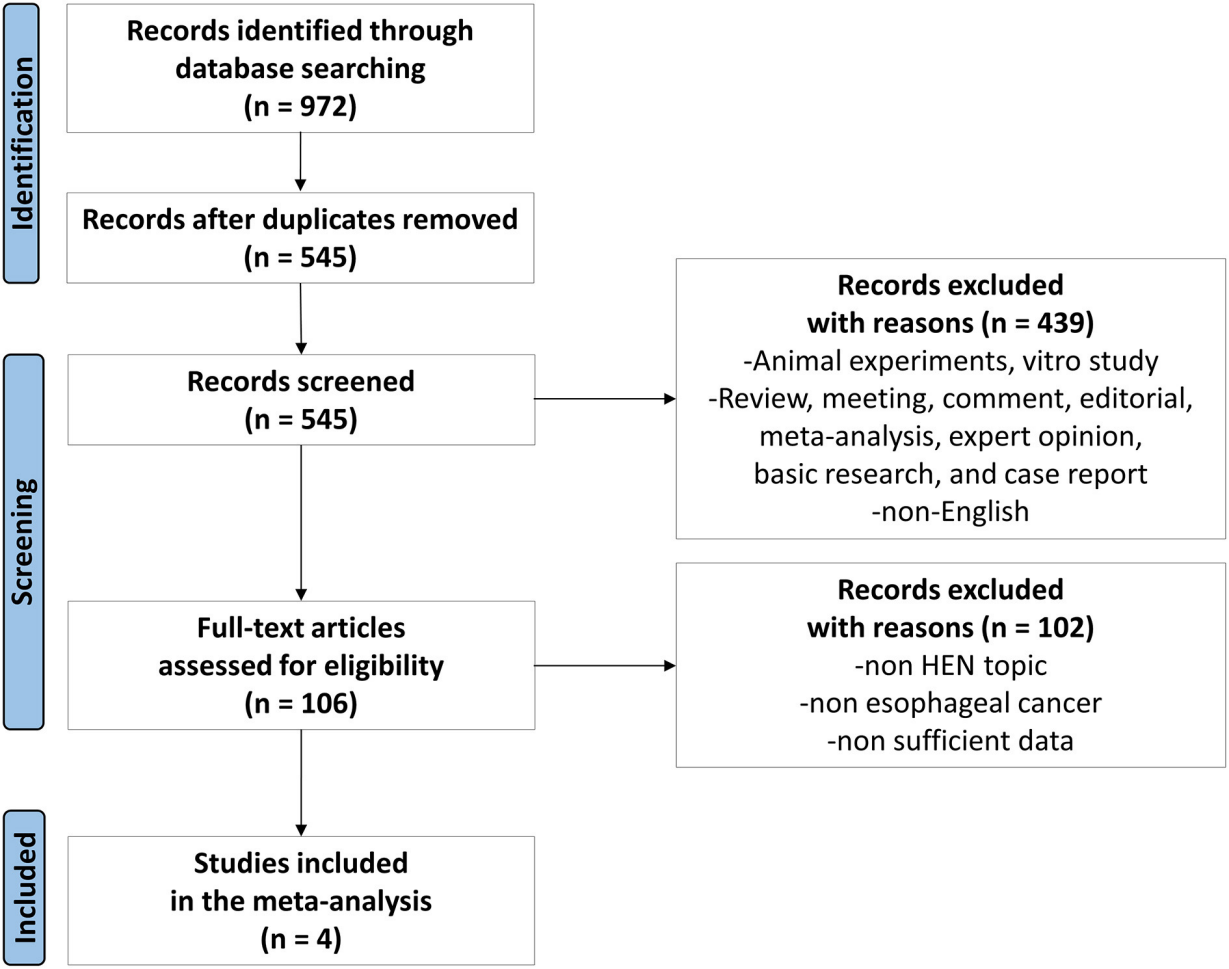
Flowchart.

All steps of the process followed the recommendations of the PRISMA 2020 flow diagram (14), and the search results have been shown in Figure 1. After an initial search, 972 articles were found through 4 databases. After the removal of 427 duplicates, then there were 439 studies removed after reviewing titles and abstracts. Later, 102 articles were found not meeting the inclusion criteria by further full-text screening. Eventually, four articles (15–18) were included for the meta-analysis (Figure 1).

### Study characteristics

This meta-analysis included four studies published between 2016 and 2020, with a total of 314 patients, including 153 patients in the HEN group and 161 patients in the NOD group. In addition to the oral diet, the HEN group was given a standard EN formula *via* a jejunostomy tube for 1–6 months, while the NOD group was only given an NOD. Most of the results were measured within 3 months. Therefore, this study only pooled the short-term results of 1–3 months. More detailed information and basic characteristics of the included studies in this meta-analysis are summarized in Table 1.

**Table 1:** Characteristics of the selected studies included in the meta-analysis.

**Study**	**Country**	**Number (male/** **female)**	**Age (mean ±SD)**	**Pathology (Squamous cell carcinoma /Adenocarcinoma/ other)**	**TNM Stage (I/II/III/IV)**	**Intervention**		**Outcome**	
						**Pathway**	**Duration**	**Time**	**Index**
Zeng et al. (15)	China	30 (24/6)	61.7 ± 8.4	30/0/0	1/13/16/0	JTF + NOD	6 months	1,4,12,24 week	MNA, QLQ-C30, QLQ-ES18
		30 (22/8)	59.3 ± 10.4	30/0/0	0/15/15/0	NOD			
Wu et al. (16)	China	67 (55/12)	62.1 ± 7.4	61/6/0	13/27/27/0	JTF + NOD	3 months	2 week, 3 month	QLQ-C30, PG-SGA, Postoperative complication
		75 (67/8)	61.1 ± 7.7	71/4/0	10/36/29/0	NOD			
Liu et al. (17)	China	26 (21/5)	62.0 ± 5.1	18/5/3	NA	JTF + NOD	1 month	1 week. 1 month	QLQ-C30, Body composition, Postoperative complication
		24 (14/10)	64.6 ± 5.9	18/5/1	NA	NOD			
Li et al. (18)	China	30 (21/9)	63.3 ± 5.2	NA	NA	JTF + NOD	1 month	0,1 month	Body composition, Immune indicators, OS, PFS
		32 (20/12)	63.6 ± 5.6	NA	NA	NOD			

*SD, standard deviation; JTF, jejunostomy tube feeding; NOD, normal oral diet; MNA, mini nutritional assessment; QLQ-C30, european organization for research and treatment of cancer (EORTC), quality of Life questionnaire-core 30; QLQ-ES18, EORTC, quality of life questionnaire-esophageal-specific module 18; PG-SGA, patient-generated subjective global assessment; NA, not available; OS, overall survival; PFS, progression-free survival*.

### Risk of bias assessment

**Figure 2:**
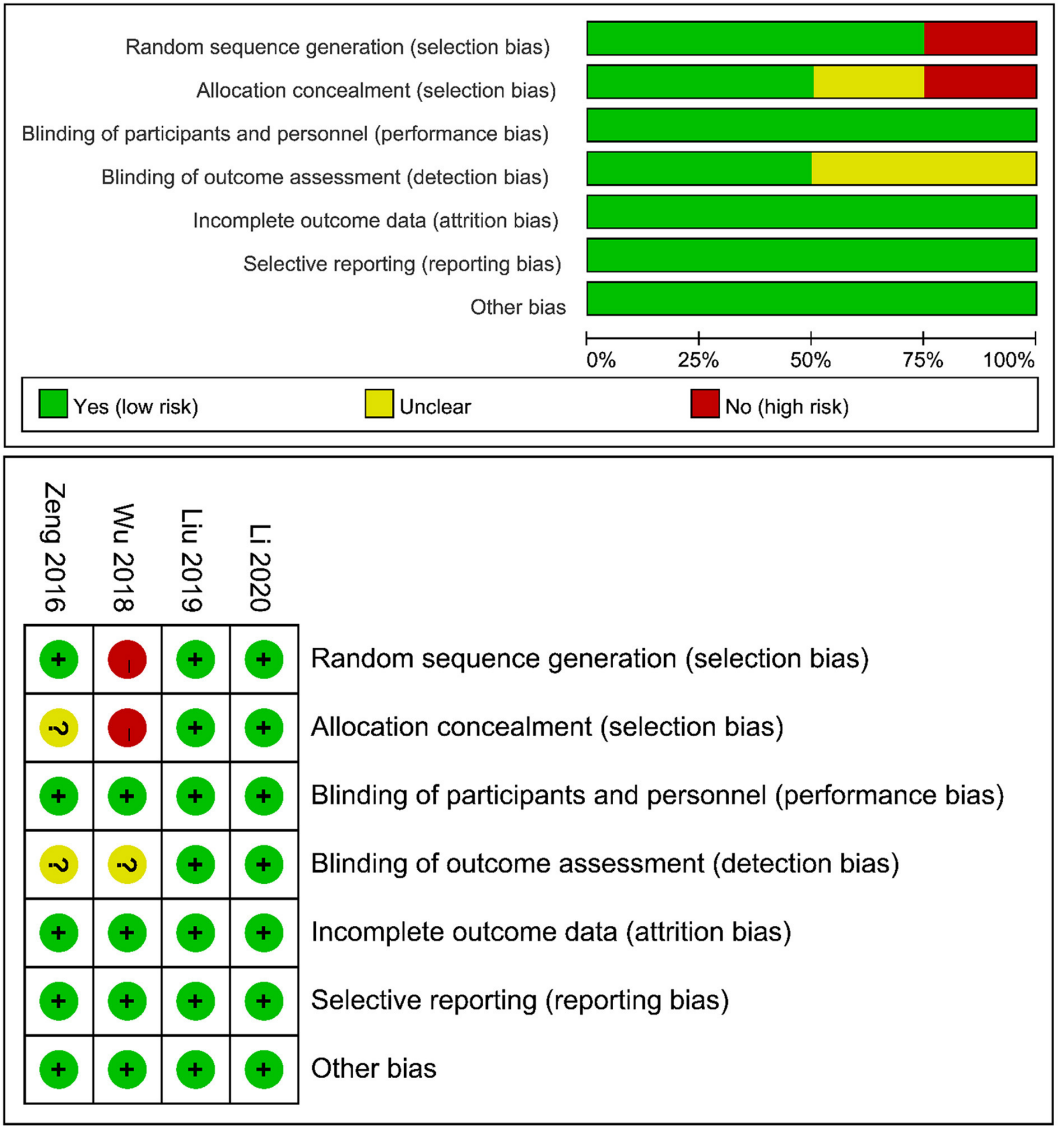
Risk of bias graph.

An overview of the risk of bias assessment following the Cochrane Library Handbook is presented in Figure 2. The risk of bias was assessed from 6 domains of bias, namely, selection bias, performance bias, detection bias, attrition bias, reporting bias, and other sources of bias. The study by Wu et al. had a potentially high risk of selection bias because the HEN group received minimally invasive esophagectomy and the NOD group received open esophagectomy. However, it was included in the analysis for no significant difference in the baseline. All trials were considered to have a low risk of performance, attrition, and reporting bias and were free of other biases. In summary, we believed that the small risk of bias among studies did not affect the results.

The pooled absolute, relative effects and quality of overall evidence supporting each outcome are shown in Table 2.

**Table 2:** Summary of pooled results and overall quality of evidence.

**Outcomes**	**Relative effect (95% CI)**	***P*-value**	**Studies**	**Patients**	**Heterogeneity**			**Publication bias**		**Quality** **of** **evidence**
					**χ^2^**	***P*-value**	***I^2^*(%)**	**Begg**	**Egger**	
Nutritional outcomes 1 month										
BMI (kg/m^2^)	WMD 0.70 (0.09, 1.30)	0.02	3	252	3.29	0.193	39.1	1	0.88	++++, high
Albumin (g/dL)	WMD 0.05 (−0.03, 0.13)	0.20	2	190	1.49	0.223	32.7	1	0.55	++++, high
Weight (kg) POD30–Preop	WMD 0.39 (−0.01, 0.79)	0.06	2	112	2.02	0.156	50.4			++++, high
LBM (kg) POD30–Preop	WMD 0.76 (0.04, 1.48)	0.04	2	101	0.20	0.652	0			++++, high
ASMI (kg/m^2^) POD30–Preop	WMD 0.30 (0.02, 0.58)	0.03	2	101	2.03	0.154	50.8			++++, high
EORTC QLQ-C30 1 month										
Physical function	WMD 5.90 (2.27, 9.53)	0.001	3	250	7.42	0.025	73	1	0.46	+++-^a^, moderate
Role function	WMD 10.32 (−6.08, 26.72)	0.22	3	250	130.21	<0.001	98.5	1	0.37	++-^b^, low
Social function	WMD 3.29 (−8.12, 14.69)	0.57	3	250	65.56	<0.001	96.9	1	0.21	++- ^b^, low
Emotional function	WMD 1.41 (−8.34, 11.16)	0.78	3	250	47.33	<0.001	95.8	1	0.36	++- ^b^, low
Cognitive function	WMD 2.88 (−3.78, 9.53)	0.40	2	190	2.73	0.099	63.3			++++, high
Fatigue	WMD −10.82 (−18.76, −2.88)	0.008	3	250	20.87	<0.001	90.4	1	0.13	+++- ^c^, moderate
Diarrhea	WMD −9.24 (−31.08, 12.60)	0.41	3	250	87.07	<0.001	97.7	1	0.27	++- ^b^, low
Pain	WMD −8.85 (−23.92, 6.21)	0.25	3	250	72.45	<0.001	97.2	1	0.81	++- ^b^, low
Dyspnea	WMD −1.09 (−7.22, 5.05)	0.73	2	190	<0.01	0.975	0			++++, high
Insomnia	WMD −10.83 (−24.76, 3.10)	0.13	2	190	3.22	0.073	68.9			++++, high
Nausea and vomiting	WMD −2.14 (−4.58, 0.30)	0.09	2	190	0.01	0.925	0			++++, high
Appetite loss	WMD −2.28 (−8.74, 4.19)	0.49	2	190	0.59	0.444	0			++++, high
Constipation	WMD 1.83 (−5.61, 9.28)	0.63	2	190	0.02	0.878	0			++++, high
Financial difficulties	WMD 6.72 (−0.37, 13.81)	0.06	2	190	0.03	0.859	0			++++, high
Global health status	WMD 1.26 (−1.06, 3.58)	0.29	2	190	0.03	0.859	0			++++, high
EORTC QLQ-C30 3 months										
Physical function	WMD 9.26 (8.00, 10.53)	<0.001	2	200	0.04	0.85	0			++++, high
Role function	WMD 9.96 (8.11, 11.82)	<0.001	2	200	1.37	0.241	27.1			++++, high
Social function	WMD 8.51 (3.48, 13.54)	0.001	2	200	4.87	0.027	79.5			+++- ^a^, moderate
Emotional function	WMD 0.37 (−9.22, 9.96)	0.94	2	200	18.22	<0.001	94.5			++- ^b^, low
Fatigue	WMD −12.73 (−14.80, −10.66)	<0.001	2	200	1.30	0.253	23.3			++++, high
Diarrhea	WMD 12.75 (−17.04, 42.54)	0.40	2	200	90.69	<0.001	98.9			++- ^b^, low
Pain	WMD −4.28 (−19.76, 11.20)	0.59	2	200	61.05	<0.001	98.4			++- ^b^, low
Postoperative outcomes										
Hospital stay (days)	WMD −0.29 (−1.76, 1.18)	0.70	3	254	1.47	0.481	0	1	0.99	++++, high
Postoperative complications	RR 1.09 (0.61, 1.94)	0.81	2	112	0.20	0.656	0			++++, high
Anastomotic leakage	RR 0.71 (0.12, 4.22)	0.70	2	194	0.07	0.797	0			++++, high
Pneumonia	RR 0.53 (0.34, 0.81)	0.004	2	192	0.57	0.449	0			++++, high

*CI, confidence interval; BMI, body mass index; POD30, postoperative day 30; Preop, preoperative; LBM, lean body mass; ASMI, appendicular skeletal muscle mass index; QLQ-C30, quality of life questionnaire-core 30; WMD, weighted mean difference; RR, risk ratios.*

a *Downgraded by one level for moderate statistical heterogeneity.*

b *Downgraded by two levels for severe statistical heterogeneity.*

c *Downgraded by one level because despite severe statistical heterogeneity, visual inspection of forest plots indicated a consistent direction in study-level treatment effects*.

### Nutritional outcomes

**Figure 3:**
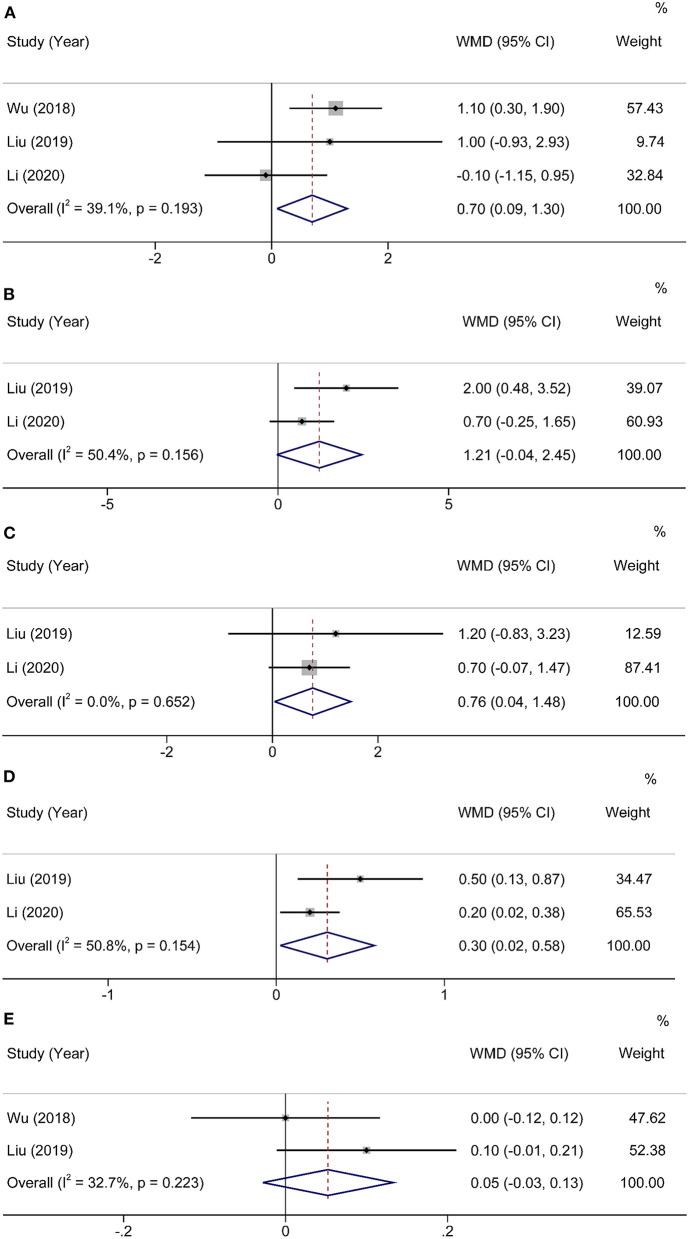
Forest plot comparing the nutritional outcomes. **(A)** BMI, **(B)** weight POD30–Preop, **(C)** LBM POD30–Preop, **(D)** ASMI POD30–Preop, and **(E)** Albumin.

To assess the impact of HEN on body mass index (BMI), a fixed-effects model was conducted to analyze since the heterogeneity was nonsignificant (χ^2^ = 3.29, I^2^ = 39.1%, *P* = 0.193). Totally, three studies (16–18) contained a number of 252 patients who compared BMI between HEN and NOD groups for 1 month after the operation, and the pooled weighted mean difference (WMD) was 0.70 (95% CI: 0.09–1.30, *P* = 0.02). Two articles (17, 18) compared the changes in body weight, lean body mass (LBM), and appendicular skeletal muscle mass index (ASMI) of patients in the HEN combined with NOD the group at 1 month after surgery. A random-effects model was used because limited studies provided this comparison. Significant statistical differences were shown in the change of LBM (WMD = 0.76, 95% CI: 0.04–1.48, *P* = 0.04) and ASMI (WMD = 0.30, 95% CI: 0.02–0.58, *P* = 0.03). HEN also showed some advantages in preventing body weight loss (WMD = 0.39, 95% CI: −0.01 to 0.79, *P* = 0.06). To investigate the effect of HEN on serological nutritional indicators for 1 month after operation, a random-effects model was used since only a limited number of albumin studies were included (16, 17). There was no significant difference in albumin between HEN and NOD groups (WMD = 0.05, 95% CI: −0.03 to 0.13, *P* = 0.20) (Figure 3).

### Quality of life

Three studies (15–17) [some domains were analyzed only in two studies (16, 17)] used the European Organization for Research and Treatment of Cancer (EORTC) general quality of life questionnaire (QLQ-C30) to evaluate QOL for 1 month after operation, and the pooled results are summarized in Table 2. Results showed that compared with the NOD group, the HEN group could improve physical function (WMD = 5.90, 95% CI: 2.27–9.53, *P* = 0.001) and reduce fatigue (WMD = −10.82, 95% CI: −18.76 to −2.88, *P* = 0.008). A random-effects model was conducted to analyze since both of the heterogeneities were significant (physical function: χ^2^ = 7.42, I^2^ = 73.0%, *P* = 0.025; fatigue: χ^2^ = 20.87, I^2^ = 90.4%, *P* < 0.001). However, there were no statistically significant differences in other domains.

**Figure 4:**
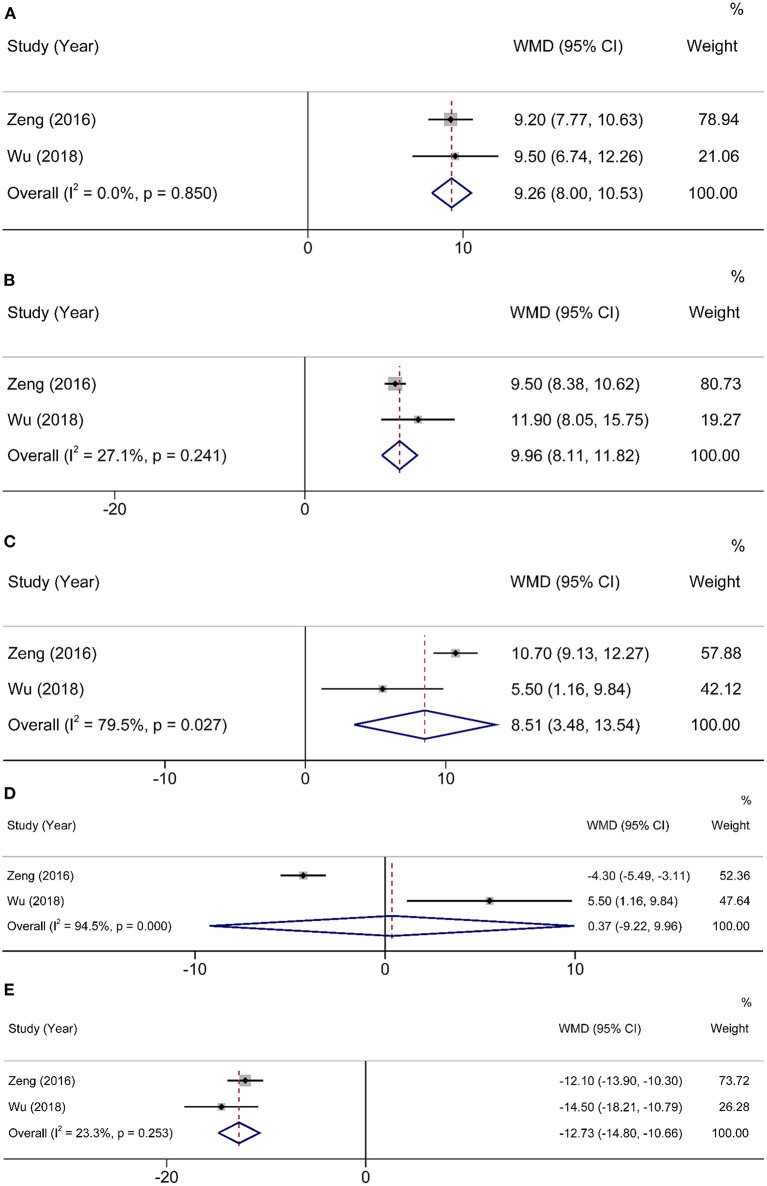
Forest plot comparing QOL at 3 months after operation. **(A)** physical function, **(B)** role function, **(C)** social function, **(D)** emotional function, and **(E)** fatigue.

Two studies (15, 16) used QLQ-C30 to assess QOL for 3 months after operation. Compared with 1 month after operation, HEN not only improved physical function (WMD = 9.26, 95% CI: 8.00–10.53, *P* < 0.001) and reduced fatigue (WMD = −12.73, 95% CI: −14.8 to −10.66, *P* < 0.001) but also improved role function (WMD = 9.96, 95% CI: 8.11–11.82, *P* < 0.001) and social function (WMD = 8.51, 95% CI: 3.48–13.54, *P* = 0.001). A random-effects model was used because only two studies were included (Figure 4).

### Postoperative outcomes

**Figure 5:**
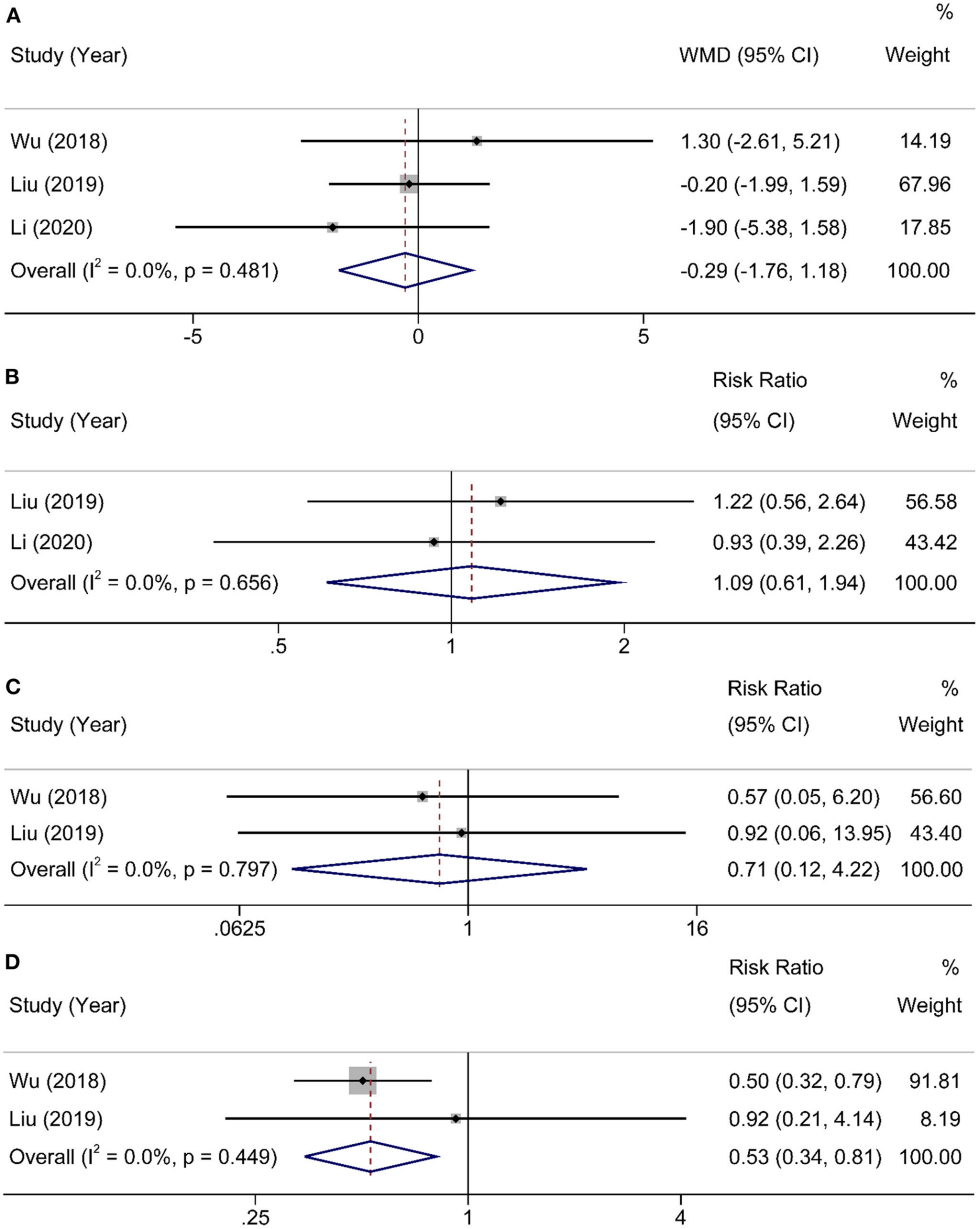
Forest plot comparing the postoperative outcomes. **(A)** hospital stay, **(B)** postoperative complications, **(C)** anastomotic leakage, and **(D)** pneumonia.

Three studies (16–18) with 254 participants provided information about postoperative hospital stays. Heterogeneity was found in studies (χ^2^ = 1.47, I^2^ = 0%, *P* = 0.481), and a random-effects model was applied to analysis. There were no significant differences in postoperative hospital stays between HEN and NOD groups (WMD = −0.29, 95% CI: −1.76–1.18, *P* = 0.70). Two articles (17, 18) studied the overall incidence of postoperative complications, and the results showed no statistical significance (RR = 1.09, 95% CI: 0.61–1.94, *P* = 0.81). The other two studies (16, 17) reported anastomotic leakage and pneumonia, and the results showed that HEN could reduce the incidence of postoperative pneumonia (RR = 0.53, 95% CI: 0.34–0.81, *P* = 0.004) but had no effect on postoperative anastomotic leakage (RR = 0.71, 95% CI: 0.12–4.22, *P* = 0.70). A random-effects model was used due to limited studies (Figure 5).

### Publication bias

Begg's and Egger's tests were used to evaluate the publication bias because <10 studies were included for analysis, and no notable publication bias was found in the meta-analysis (Table 2).

## Discussion

Esophageal cancer is a common malignant tumor of the gastrointestinal tract with a poor prognosis. Esophagectomy is the cornerstone of multimodal treatment strategy for locoregional EC (19). No matter which surgical method is used, postoperative gastrointestinal symptoms, including early satiety, dysphagia, postprandial dumping syndrome, and reflux, usually result in nutritional intake problems (20). Therefore, the nutritional status and QOL of most patients with EC will deteriorate significantly after operation. Nearly two-thirds of patients who underwent esophagectomy lose more than 10% of their preoperative BMI for 6 months after discharge (21). Studies have confirmed that weight loss is an independent risk factor for increasing complications and reducing survival (22, 23). Therefore, early postoperative nutritional support is necessary for patients undergoing esophagectomy. HEN is a relatively new nutritional intervention that provides patients with EN support at home through jejunostomy or nasogastric feeding tubes. To the best of our knowledge, this systematic review and meta-analysis is the first study of the effect of HEN on postoperative patients with EC. In addition, the results showed that HEN is of great significance in improving the QOL and reducing complications in patients with EC following an esophagectomy.

Our meta-analysis, including four studies with 314 patients, illustrated that HEN has a favorable impact on postoperative BMI, LBM, and ASMI. Moreover, HEN also shows advantages in preventing weight loss. The most common manifestations of malnutrition are weight loss and decreased albumin. Malnutrition is generally considered to occur when patients lose weight significantly and is usually ignored in normal weight or obese populations. For these people, LBM can better reflect their nutritional status (24). However, our results did not find a difference in postoperative albumin between HEN and NOD groups.

The EORTC QLQ-C30 has been widely used to assess the postoperative functions and symptoms. A large number of studies have shown that QLQ-C30 objectively reflects the QOL of all types of patients with cancer (25). Poor QOL has been shown to be an independent negative prognostic factor in patients with gastro-esophageal cancer (26). From the meta-analysis, we found that physical function, role function, and social function of the HEN group were better than those of the NOD group, and HEN could reduce the fatigue of patients. These results are more obvious at 3 months after operation than at 1 month after operation. Smalley et al. (27) found that the completion of cancer treatment was significantly associated with good physical function. HEN can improve the physical function of patients and relieve fatigue, contributing to the completion of cancer treatment.

We also found that HEN reduced the incidence of postoperative pneumonia, but there was no notable difference in the overall incidence of complications and anastomotic leakage between the two groups. The reason may be that the vast majority of anastomotic leakage occurred in the early postoperative period, so HEN had less effect on it after discharge.

Li et al. (18) also found that HEN improved the immune function of patients with EC. The increasing levels of natural killer (NK) cells, immunoglobulin (Ig)G, and IgA in the HEN group were significantly higher than that in the NOD group. NK cells play an important role against tumor cells by expressing activating and inhibitory receptors recognized by tumor cells (28). High levels of IgG reduce the risk of early tumor metastasis and the incidence of infectious complications after discharge. Secretory IgA is the main antibody of mucosal infection. The increase in IgA level can help to maintain the integrity of intestinal mucosal cells' structure and function and protect the intestinal mucosal barrier (29, 30). Therefore, HEN may be beneficial to patients with EC by improving their immune status. However, no studies have investigated the mechanism by which HEN improves immune function, which needs more studies to confirm this finding.

The effect of HEN on long-term prognosis has only been studied in two articles. The results of Lorimer et al. (31) showed that the 90-day mortality rate decreased significantly in the HEN group (12.2 vs. 15.8%, *P* = 0.016). Li et al. (18) studied the 2-year progression-free survival (PFS) and overall survival (OS) but found no significant difference (log-rank, 0.36 and 0.29, respectively). Although this study did not analyze long-term results, it has been demonstrated that the results (body weight, BMI, and QOL) improved by HEN are closely related to OS (32).

This analysis had several limitations. First, all the studies included were from China. Second, the total amount of patients in the studies was only 314, and the remaining patients suffer from limited sample size. Third, insufficient studies have analyzed the impact of HEN on long-term outcomes such as OS, PFS, recurrence-free survival (RFS), and disease-free survival (DFS). Thus, more well-conducted studies with large sample size were urgently needed to confirm and update our conclusion. Meanwhile, the following studies should complete long-term survival outcomes, and patients from different races should also be included.

## Conclusion

Home enteral nutrition improved nutritional status and QOL in postoperative patients with EC and reduced fatigue and the incidence of postoperative pneumonia. All in all, the results of our meta-analysis support the use of HEN after esophagectomy. Meanwhile, more high-quality studies are needed to confirm the findings.

## Data availability statement

The original contributions presented in the study are included in the article/supplementary material, further inquiries can be directed to the corresponding author.

## Author contributions

CZha and ZZ-C conceptualized the study, revised the manuscript, and supervised the study. LW-H and YQ conceptualized the study, drafted the manuscript, and made the figures. CZhe, ZZ-C, and CL collected the literature and revised the manuscript. All authors contributed to the article and approved the submitted version.

## Funding

This study was supported by the National Natural Science Foundation of China [81702444] and the Natural Science Foundation of Jiangsu Province [BK20181239].

## Conflict of interest

The authors declare that the research was conducted in the absence of any commercial or financial relationships that could be construed as a potential conflict of interest.

## Publisher's note

All claims expressed in this article are solely those of the authors and do not necessarily represent those of their affiliated organizations, or those of the publisher, the editors and the reviewers. Any product that may be evaluated in this article, or claim that may be made by its manufacturer, is not guaranteed or endorsed by the publisher.

## Abbreviations

EC: Esophageal cancer
QOL: Quality of life
PN: Parenteral nutrition
EN: Enteral nutrition
HEN: Home enteral nutrition
NOD: Normal oral diet
BMI: Body mass index
LBM: Lean body mass
ASMI: Appendicular skeletal muscle mass index
OS: Overall survival
PFS: Progression-free survival
RFS: Recurrence-free survival
DFS: Disease-free survival.
